# Monitoring utilisation patterns of home- and community-based services for healthy ageing: a cross-sectional study

**DOI:** 10.1186/s12877-025-05918-5

**Published:** 2025-04-28

**Authors:** Wusi Zhou, Xiangjing Zhang, Xiaoyang Lyu

**Affiliations:** 1 School of Public Administration, Hangzhou Normal University, Hangzhou, China; 2 Fudan Institute on Aging, Fudan University, Shanghai, China

**Keywords:** Community care, Service utilisation, Ageing in place, Older people, Healthcare policy

## Abstract

**Background:**

The home- and community-based care model has been developed to enable older people healthy ageing in place. However, few studies have investigated how well particular home- and community-based services (HCBS) have reached older people. This is crucial to address the issue of inequity in healthcare for seniors living independently. This study aims to examine the utilisation of the overall HCBS as well as its three main categories: life care services (LCS), medical health services (MHS) and spiritual comfort services (SCS). It will compare various predictors influencing the uptake of these services and highlight broader policy implications for building an integrated care system that promotes ageing within communities.

**Methods:**

The research adopted a cross-sectional research design with a comparative analysis approach. A questionnaire survey was carried out with 1246 older adults in Zhejiang province, yielding 1171 valid responses and an overall response rate of 93.98%. Descriptive statistics and binary logistic regression analyses were applied to identify differences in service utilisation and key determinants.

**Results:**

The research findings showed that overall HCBS utilisation was relatively low, influenced by factors such as age, family support, self-assessed economic status, community type, type of social security benefits, awareness of regular HCBS, self-care capability and self-rated health. Substantial variations were observed in the utilisation rates of LCS, MHS and SCS. The young-old population was less likely to use LCS and MHS, whereas individuals with family support and chronic diseases were more inclined to utilise MHS and SCS. Economic condition and community type emerged as key determinants affecting the use of all three services.

**Conclusions:**

This study highlights that HCBS have not been fully utilised and are influenced by various factors. Moving forward, it is crucial to address the care needs of different income groups, with particular attention to the unique challenges faced by rural older adults. Targeted policies and interventions are necessary to enhance accessibility for those with chronic illnesses and limited family support. Local governments should work in partnership with families to develop higher-quality, place-based integrated care services, ensuring that both medical care and spiritual well-being services are effectively delivered to meet the evovling needs of an ageing population.

## Introduction

Population ageing has become a global phenomenon since 1970s. Rather than moving to nursing homes, older people generally prefer to live independently in their own houses for as long as possible [1]. A study of OECD countries showed that 50%-70% of older adults preferred to receive home- and community-based services (HCBS) for independent living at home [2]. HCBS is defined as any form of supportive care, including physical, medical and spiritual activities, that provided by professional caregivers in the home or community to assist older people ageing in place. It fully leverages the strengths of family and community, with the family serving as the foundation of HCBS and the community providing necessary care for older adults in need [3, 4]. Accordingly, national, provincial and local governments introduced policies to develop HCBS programmes for supporting older people to regain their independence and achieve the highest possible quality of life [5, 6]. For example, the UK government issued policies like *Opportunity Age* to offer community care, including housing-related services, financial support and professional care services, with the purpose of promoting greater independence among older people. Similarly, the US government launched programmes, such as *Community-based Support and Services* [7] *and Home and Community Based Services* [8], to meet the needs and preferences of older adults ageing at home in later life. Despite this, HCBS remained underutilised in many countries, with significant disparities in service usage across different regions. In Carsyn’ study, only 10% of older adults received home services, 13% applied for financial assistance, 18% used community services, and another 42% accessed to other services [9]. Multiple factors have been identified as barriers to the utilisation of HCBS. Stoddart reported that the use of these services was influenced by a variety of factors, such as functional ability, emotional well-being, cognitive impairment, foot problems, and the frequency of fall [10]. Stoller identified that older adults with sufficient financial resources or lived alone with activities of daily living issues were more likely to use HCBS [11]. 

In China, the rapid ageing of the population, coupled with shrinking households and large-scale migration, has made traditional family-based care no longer sufficient to meet the growing needs for elderly support. In response, developing home-based integrated services has emerged as a national policy priority. At the national level, the *Opinions on Providing Community Care Services for Ageing at Home* was issued to define HCBS as providing life care, housekeeping services, rehabilitation and nursing care and spiritual comfort for older adults living independently at home [12]. This was followed by several key policies, such as the *Opinions on Promoting the Development of Elderly Care Services* and the *Opinions on Promoting the Construction of the Basic Elderly Care System*, which further emphasized the fundamental role of HCBS and outlined a national framework for basic elderly care services, covering material assistance, caregiving services and emotional support [13]. To enhance access to HCBS, provincial governments have implemented various strategies and guidelines to improve service provision. For example, Zhejiang Province approved the *Implementation Opinions on Accelerating the Development of Elderly Care Service Industry* to ensure comprehensive HCBS coverage across all towns. Additionally, the *Promoting High-quality Development and Construction of “Health Care in Zhejiang” Work Plan* (2022–2025) set five key goals of offering support, medical care, action, learning and enjoyment for older people [14]. At the local level, cities such as Hangzhou have published policies like the *Regulations on HCBS* to establish a community-based care network for seniors ageing at home. As a result, the number of community-based care organizations expanded significantly from 18,900 in 2014 to 291,000 in 2020. Similarly, the avaiability of care beds increased from 1.875 million to 3.328 million [15]. Key HCBS, such as dainly care, meal preparation, bath help, emergency visit, medical assistance, psychological counseling and recreational activities, have become more widely accessible [16]. These services can be broadly classified into three primary categories of life care services (LCS), medical health services (MHS) and spiritual comfort services (SCS). Housekeeping services were not designated as a separate category, because they closely overlap with LCS and are often provided together in practical service delivery. Housekeeping tasks, such as cleaning, laundry and meal preparation are fundamental to maintain daily living and align with the core functions of LCS, which focus on assisting older adults with personal care, mobility and basic daily activities. As Wang et al. emphasized, home-based elderly care services operate within a more limited scope than institutional care, primarily addressing life care, medical services, and spiritual well-being [17]. Similarly, Cui et al.’s study found that LCS, MHS and mental & cultural services were the most commonly provided at the local level, accounting for 79.2%, 89.6% and 89.4% respectively [18]. This study adopted the same classification, grouping HCBS into three key service categories. LCS encompass community-based assistance with daily activities for older people, including meal preparation, bathing, cleaning and walking services (e.g., Fu and Chui [19]; Zhou et al. [20]). MHS focus on health maintenance, incorporating services such as home medical care, rehabilitation nursing, assistive device rentals and health education (e.g., Wang et al. [21]). SCS cater to the spiritual and emotional needs of older adults by providing psychological support, social interaction, and recreational activities (e.g., Dai et al. [22]).

## Methods

### Andersen behavioral model

Based on the research’s objectives, a quantitative approach was employed to investigate HCBS utilization, applying the Andersen Behavioral Model to identify key elements that influence service utilization patterns. This model, originally proposed in 1968 by Ronald Andersen [23, 24], seeks to understand how and why individuals use healthcare services and to inform policies that promote equitable access and utilisation. It has been widely applied in medicine and public health research globally (e.g., Peng, Song and Huang [25]; Travers, Hirschman and Naylor [26]). Although the Andersen behavioral model has undergone five modifications, it continues to rely on its three core factors of predisposing, enabling, and need to explain utilisation behavior across different groups. Predisposing factors refer to personal characteristics, such as demographics, attitude or willingness to seek healthcare services. Enabling factors are related to the availability and accessibility of family and community resources. Need factors reflect an individual’s perceived or actual health need, including their health status and functional abilities (Li et al. [27]).

**Fig. 1:**
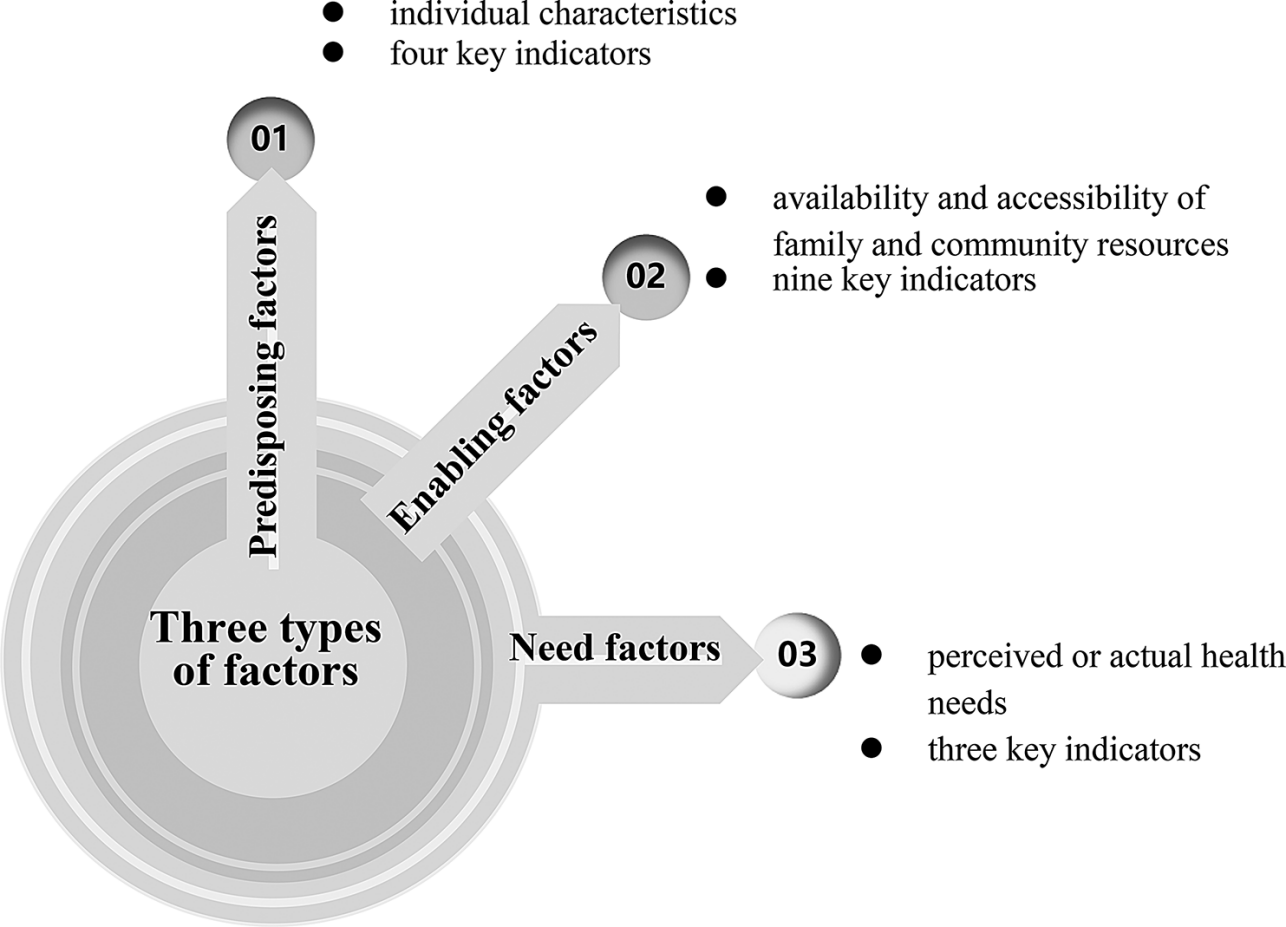
Three types of factors associated with the utilisation of HCBS

Correspondingly, this study defines HCBS utilisation as a function of an individual’s predisposing, enabling and need factors, which influence their access to services (Fig. 1). Predisposing factors are associated with individual characteristics that affect the likelihood of using HCBS, regardless of personal condition. These factors are represented by four key indicators of gender, marital status, age and educational attainment that affect the likelihood of using HCBS, regardless of personal condition. Enabling factors primarily determine HCBS accessibility and include nine indicators: family support, live with children, self-assessed economic status, community type, social security benefits, perception of HCBS provision, perception of LCS provision, perception of MHS provision, and perception of SCS provision. Need factors are represented by three indicators, such as chronic illness, self-care capability and self-rated health, which directly influence the necessity of HCBS utilization [28]. 

### Research design

This study adopted a cross-sectional quantitative design to assess differences in the utilisation of both the overall HCBS and its three main categories of LCS, MHS and SCS. A questionnaire survey on HCBS was carried out across five town streets, including HM, BY, HJ, SQ and DC, located in JJ district, Taizhou city, Zhejiang province. Four reasons led to this choice. First, Taizhou has been a national frontrunner in implementin HCBS policies and projects in response to government guidelines. The local government has actively established HCBS centers that provide meal assistance, bathing services, entertainment, mental support and medical care for older adults. Secondly, JJ District has experienced a fast pace of population ageing, which prompted the local government to expand regular and on-call HCBS as part of a broader effort to reform the community care system. As a result, JJ District has been selected as a representative sample site for major national studies, including both the fourth national survey and the Monitoring Survey on the Living Conditions of Urban and Rural older people in China. Thirdly, the degree of population ageing varied across the five town streets. In QS and BY, older adults made up less than 20% of the population and was positioned at the early-stage of ageing, while in DC, HJ and HM the proportion exceeded 20% and was in state of the middle-stage of ageing. Besides, the local authorities in all five town streets actively supported the study by assisting researchers in accessing older people for face-to-face surveys.

### Data collection

The survey questionnaire consists of 35 questions, covering individual characteristics, health condition, living status, the use of HCBS, LCS, MHS and SCS. A stratified and systematic sampling method was employed. First, the sample size for urban and rural areas, as well as the proportion of older adults across different age groups, were determined based on the age and urban-rural distribution of the older population in the entire district. Second, a total of 15 communities were selected as survey sites, including three urban communities chosen from each street of HM and BY, and three rural communities from each street of HJ, QS, and DC. Third, starting from the first community, all eligible survey participants were listed by age and then randomly selected until the required sample size was reached. Prior to the main survey, a pilot study was conducted with 32 older adults in DC Town. As a result of this pilot, modifications were made to some questions, in order to improve comprehensibility and accuracy. The finalized questionnaire was administered by trained voluntary workers, who conducted face to face surveys with a random sampling of 1246 older people between May and July 2021. After excluding incomplete and inconsistent responses, 1171 valid responses were collected, resulting in an overall response rate of 93.98%, as shown in Table 1.

**Table 1:** Different levels of survey responses in six town streets

Town Street	Survey Samples		Valid Samples	
	Number	Percentage	Number	Percentage
HM	340	27.29%	320	25.68**%**
BY	311	24.96%	293	23.51**%**
HJ	235	18.86%	223	17.90**%**
SQ	232	18.62%	216	17.34**%**
DC	128	10.27%	119	9.55**%**
Total	1246	100.00%	1171	93.98**%**

### Data analysis

#### Dependent variable

The dependent variables refer to the utilisation of both overall HCBS and its three service categories of LCS, MHS and SCS. Receiving at least one of HCBS was coded as “1” (“0” otherwise). In terms of LCS, using any of services like meal, bathing, cleaning and walking services was assigned a value of “1” (“0” otherwise). As for MHS, utilising any of services such as home medical care, home care bed, rehabilitation nursing, assistive device rental and health education was assigned a value of “1” (“0” otherwise). Regarding SCS, receiving psychological support or chat service was coded as “1” (“0” otherwise).

### Independent variables

The independent variables present a view of predisposing, enabling and need factors. Predisposing factors include four key indicators. “Male” and “Married” was coded as “1”, compared with “0” for “female” and “not married (divorced, widowed, and unmarried)”. Age was categorised into three groups of 60–69 years (“1”), 70–79 years (“2”) and ≥ 80 years (“3”). Education attainment was also classified into three levels as “primary school and below” (“1”), “middle and high school” (“2”) and “tertiary institutions degree and above (vocational high school)”(“3”).

Enabling factors were measured by using nine indicators, each assigned specific values for analysis. “Family support” and “Living with children” were coded as “1” if present (“0” otherwise). “Rural community” and “Urban employees’ basic endowment insurance” were assigned a value of “1”, whereas “0” was for “urban community” and “urban-rural residents’ endowment insurance”. Self-assessed economic status was rated on a 1–3 point scale, with 1 representing “poor” and 3 indicating “wealthy”. Perception of LCS provision, reflecting older adults’ awareness of services such as meal, cleaning, bathing and walking assistance, was coded as “1” if perceived as available (“0” otherwise). Similarly, prerception of MHS provision, referring to awareness of services including health education, home medical care, rehabilitation nursing and assistive device rental, was similarly coded as “1” if available (“0” otherwise). Perception of SCS provision, measuring awareness of services like psychological support and chat service, was assigned “1” for perceived availability (“0” otherwise).

Need factors cover three indicators. Individuals with a chronic disease was given a value of “0”, or “1” otherwise. Self-care capability was measured by activities of daily living (ADLs), which include bathing, dressing, toileting, transferring, continence and feeding. Older adults with “Full self-care capability”, meaning they could independently perform all six ADLs, was assigned a value of “1”, while “0” was given to those with “impaired self-care capability” who were unable to complete one or more of these activities. Self-rated health was evaluated on a 3-point scale, with 1 representing “unhealthy”, 2 for “average”, and 3 indicating “healthy”.

### Statistical analyses

Descriptive statistics were used to summarize and present the key characteristics of the data, which provided an overall picture of the sample and helped to identify any notable patterns in service utilisation. To assess the relationships between different factors and service utilisation, the Spearman correlation coefficient test was then conducted with a significance level set at *P* < 0.05. The results indicated that the absolute values of the Pearson correlation coefficients between independent variables in all four models remained below 0.6. Furthermore, the tolerance values of the independent variables in all four models were greater than 0.5, and the Variance Inflation Factor (VIF) values were less than 5. This confirmed that there was no significant multicollinearity between the independent variables, ensuring the robustness of the regression analysis and the reliability of the estimated relationships between predictors and HCBS utilization.

Since all independent variables are dichotomous, binary logistic regression was applied to analyse the multiple important factors that influence HCBS utilisation. This method allows for estimating the probability of service utilization based on various predisposing, enabling, and need factors. An empirical model was developed as follows:

$$\:\text{ln}\left(\frac{{\text{p}}_{\text{i}}}{1-{\text{p}}_{\text{i}}}\right)={\upalpha\:}+{\upbeta\:}{\text{X}}_{\text{i}\text{j}}+{\upepsilon\:}$$

Where pi represents the probability that the ith older adult utilise any of HCBS; X_ij_ denotes the independent variables, which include predisposing, enabling and need factors. β is the coefficient matrix; α is the constant term, and ε denotes the error term, capturing the portion of the dependent variable that cannot be explained by the independent variable. The coefficient β describes the strength and direction of relationship between two variables and whether their associations are statistically significant. Four binary regression analysis models of M1 to M4 are conducted to determine the factors that influence HCBS utilisation, including overall HCBS (M1) and its three typical categories of LCS (M2), MHS (M3) and SCS (M4). The chi-square values for these models were 341.092 (M1), 333.092 (M2), 268.993 (M3) and 285.792 (M4), all of which reach a significance level of *p* < 0.001. To assess the goodness of fit of the models, the Hosmer-Lemeshow test was performed, and the results for all four models showed *p* > 0.05, indicating that the models fit the data well and that the independent variables provide valuable insights to HCBS utilisation.

## Results

Table 2 presents the descriptive statistics of the dependent variables that measure respondents’ utilisation of overall HCBS as well as its three categories of LCS, MHS and SCS. Among the 1171 older people surveyed, 32.45% has used at least one of HCBS. However, significant differences were observed in the utilisation rates across the three service categories. MHS were reported the highest utilisation rate at 26.73%, compared with 13.15% for LCS and 7.34% for SCS. To be specific, health education was the most commonly used service, utilised by 21.01% of the older population. By constrast, the utilisation rates for other professional services remained below 10%. Notably, fewer than 5% of older people had used services such as walking assistance, rehabilitation nursing, assistive device rental, or psychological support.

**Table 2:** Descriptive statistics of dependent variables

Type of Services	Number	utilisation Rate (%)
Life care services	154	13.15
Meal service	106	9.05
Cleaning service	96	8.20
Bathing service	85	7.26
Walking service	39	3.33
Medical health services	313	26.73
Health education	246	21.01
Home medical care	108	9.22
Rehabilitative nursing	54	4.61
Assistive device rental	45	3.84
Spiritual comfort services	86	7.34
Psychology support	25	2.13
Chat service	75	6.40
Total	380	32.45

Table 3 presents the descriptive statistics of the independent variables, including predisposing, enabling and need factors that influence HCBS utilisation. As for predisposing factors, 60.55% of older respondents were female, reflecting the gender disparity in life expectancy and the female advantage in longevity. Marriage was common among older adults, with 75.41% being married. 46.80% of participants were in the 60–69 age group, followed by 36.29% in the 70–79 age group and 16.91% aged 80 and above. Educational attainment was general low, as 73.01% of seniors had only primary school education or below, indicating very limited formal education among the older population. With regard to enabling factors, due to the ongoing trend of family miniaturization and nucleation in China, many older people lived independently (68.83%) rather than with family members. 73.44% relied on their own income rather than depending on their children. The majority of older people described their financial situation as “average” (63.45%) or “wealthy” (28.53%), with an average self-assessed economic level of 1.7950. 52.35% of older adults resided in urban communities, while 31.51% were covered by urban employees’ basic endowment insurance. Perceptions of social service provisions were relatively low, with 44.66% reporting yes for HCBS, 39.71% for LCS, 29.04% for MHS and 17.85% for CSC. In terms of need factors, 75.15% of older adults suffered from at least one chronic illness, while 91.80% reported to have the ability to complete ADLs and support self-care. Self-rated health was generally positive, with 64.39% of respondents rating their health as good, compared with only 4.53% who considered themselves unhealthy.

**Table 3:** Descriptive statistics of independent variables

Independent variable	Coding	Percentage (%)	Mean	SD	Min	Max
**Predisposing factors**						
Gender	0 = female 1 = male	60.55 39.45	0.3945	0.4890	0	1
Marital status	0 = Not Married 1 = Married	24.59 75.41	0.7541	0.4308	0	1
Age	1 = 60–69 2 = 70–79 3 ≥ 80	46.80 36.29 16.91	1.7011	0.7404	1	3
Educational attainment	1 = Primary school and below 2 = middle and high school 3 = Tertiary institutions degree and above	73.01 23.65 3.33	1.3032	0.5274	1	3
**Enabling factors**						
Family support	0 = No 1 = Yes	73.44 26.56	0.2656	0.4418	0	1
Live with children	0 = No 1 = Yes	68.83 31.17	0.3117	0.4634	0	1
Self-assessed economic status	1 = poor 2 = average 3 = wealthy	8.03 63.45 28.53	1.7950	0.5690	1	3
Community type	0 = Rural community 1 = Urban community	47.65 52.35	0.5235	0.4997	0	1
Social security benefits	0 = Urban-rural residents 1 = Urban employees	68.49 31.51	0.3151	0.4648	0	1
Perception HCBS provision	0 = No 1 = Yes	55.34 44.66	0.4466	0.4974	0	1
Perception of LCS provision	0 = No 1 = Yes	60.29 39.71	0.3971	0.4895	0	1
Perception of MHS provision	0 = No 1 = Yes	71.96 29.04	0.2904	0.4541	0	1
Perception of SCS provision	0 = No 1 = Yes	82.15 17.85	0.1785	0.3831	0	1
**Need factors**						
Chronic illness	0 = Yes 1 = No	75.15 24.85	0.7515	0.4323	0	1
Self-care capability	0 = impaired self-care 1 = full of self-care	8.20 91.80	0.9180	0.2745	0	1
Self-related health	1 = unhealthy 2 = average 3 = healthy	4.53 31.08 64.39	2.5986	0.5754	1	3

Table 4 displays the results of binary regression models (M1 to M4) that underline the correlations between independent and dependent variables. Specifically, M1 incorporated all predisposing, enabling and need factors to measure their effects on the overall utilisation of HCBS. The results revealed that among the predisposing factors only the 60–69 age group was a significant predictor of HCBS utilisation. Indeed, the utilisation rates for the youngest-old and middle-old groups were just 52.6% and 66.1% of the rate observed in the oldest-old group. Among enabling factors, four variables of family support, self-assessed economic status, community type and perception of HCBS provision remained significant predictors of HCBS utilisation. Older people who received support from their children or relatives were 1.510 times more likely to use HCBS compared to others without family support. Conversely, living with children was not a significant predictor of HCBS utilisation. There was a negative correlation between self-assessed economic status and service.

**Table 4:** Model analysis of factors to the utilisation of community home-based care services

Variables	M1				M2				M3				M4			
	B	exp (B)	95%CI for OR		B	exp (B)	95%CI for OR		B	exp (B)	95%CI for OR		B	exp (B)	95%CI for OR	
			Lower limit	Upper limit			Lower limit	Upper limit			Lower limit	Upper limit			Lower limit	Upper limit
**Predisposing factors**																
Gender (female)	0.89	1.093	0.800	1.493	-0.047	0.954	0.394	0.993	-0.020	0.980	0.596	1.125	-0.014	0.986	0.510	1.905
Marital (Not married)	-0.125	0.883	0.613	1.271	0.185	1.203	0.702	2.063	-0.194	0.824	0.568	1.195	-0.025	0.975	0.483	1.971
Age (≥ 80)																
Lower-age(60–69)	-0.643	0.526^**^	0.340	0.812	-1.375	0.253^***^	0.140	0.458	-0.502	0.605^*^	0.391	0.937	-0.253	0.776	0.338	1.786
Middle-age(70–79)	-0.415	0.661	0.427	1.022	-1.363	0.256^***^	0.142	0.461	-0.285	0.752	0.483	1.171	-0.168	0.183	0.380	1.881
Educational attainment (Tertiary institutions degree and above)																
Primary school and below	0.521	1.684	0.579	4.900	0.790	2.203	0.505	9.659	0.433	1.542	0.537	4.426	-0.526	0.591	0.117	2.977
Middle and high school	0.486	1.620	0.559	4.731	1.024	2.785	0.628	12.349	0.388	1.474	0.512	4.248	-0.173	0.841	0.172	4.107
**Enabling factors**																
Family support (No)	0.412	1.510^*^	1.080	2.111	0.179	1.196	0.734	1.948	0.697	2.009^***^	1.444	2.791	0.971	2.640^**^	1.388	5.022
Live with children (No)	-0.300	0.741	0.536	1.024	-0.397	0.670	0.414	1.091	-0.298	0.743	0.529	1.042	-0.761	0.467	0.235	0.928
Self-assessed economic status (pool)																
Average Wealthy	-0.716 -1.016	0.489^**^ 0.362^***^	0.290 0.199	0.825 0.658	-0.860 -1.160	0.432^*^ 0.314^**^	0.207 0.140	0.864 0.704	-0.290 -0.342	0.749* 0.710*	0.434 0.382	1.290 1.322	-0.925 -1.171	0.396^*^ 0.396^*^	0.160 0.106	0.981 0.911
Community type (Rural community)	-1.510	0.221^***^	0.150	0.325	0.858	2.359^**^	1.408	3.949	-2.008	0.134^***^	0.087	0.208	-1.170	0.310^**^	0.150	0.642
Social security benefits (Urban-rural residents’ basic endowment social security)	0.300	1.350	0.836	2.110	0.825	2.281^**^	1.316	3.958	-0.566	0.761^**^	0.346	0.932	0.744	2.104	0.950	4.663
Perception HCBS provision (None)	2.154	8.619^***^	6.238	11.910												
Perception LCS provision (None)					3.795	44.471^***^	21.411	92.396								
Perception MHS provision (None)									1.634	5.122^***^	3.644	7.207				
Perception SCS provision (None)													4.597	99.209^**^	43.802	224.600
**Need factors**																
Chronic illness (Yes)	-0.318	0.727	0.703	0.753	-0.470	0.625	0.367	1.063	-0.417	0.659^*^	0.459	0.947	-0.982	0.374^*^	0.170	0.827
Self-care capacity (Impaired self-care)	-0.683	0.505*	0.296	0.863	-0.792	0.453*	0.229	0.894	-0.411	0.663	0.383	1.148	-0.930	0.394	0.153	1.017
Self-related health (Poor)																
Average	-0.759	0.468^***^	0.334	0.657	-0.457	0.633	0.234	1.710	-0.919	0.399^***^	0.278	0.572	-0.223	0.250	0.426	1.504
Good	-1.314	0.269^**^	0.118	0.615	-0.387	0.472	0.431	1.073	-1.147	0.318^*^	0.131	0.772	-1.408	0.245	0.058	1.025
Constant	0.761	2.140			-3.436	0.032			-0.326	0.722			-0.115	0.891		
Sample size	1171				1171				1171				1171			
Nagelkerke R2	0.353				0.458				0.299				0.530			

Reference groups in parentheses; *, **, and *** indicate significance at the 5%, 1%, and 0.1% levels, respectively

Utilisation, as those with higher income presented a lower proportion of HCBS usage. In addition, respondents residing in urban communities were less likely to use HCBS than their rural counterparts. Higher awareness of service availability was strongly associated with greater utilisation of HCBS. Among the need factors, both self-care capability and self-rated health were significantly correlated with HCBS utilisation. Particularly, older adults with full self-care capability reported lower HCBS utilisation than those with impaired self-care capability. Furthermore, older people with poor self-rated health demonstrated a greater inclination towards HCBS utilisation compared to their healthier counterparts.

M2 examines the association of predisposing, enabling and need factors with the current utilisation of LCS. As shown in the predisposing factors, age was the only siginificant determinant of service utilisation, with older adults in the 60–69 age group being less likely to use LCS than those in the 70–79 and ≥ 80 age groups. Several enabling factors, including self-assessed economic status, community type, social security benefits and perception of LCS provision, were significantly associated with LCS utilisation. To be specific, lower-income individuals exhibited a stronger incentive to participate in LCS projects. Likewise, those who lived in urban communities or enjoyed the urban employees’ basic endowment insurance reported higher levels of LCS utilisation. Awareness of LCS played a crucial role in utilisation, as those who were aware of LCS were over 44 times more likely to use them compared to those without any knowledge. In terms of the need factors, self-care capability was the only significant predictor of LCS utilisation. Respondents with self-care deficits were more likely to use LCS than those with full self-care capability.

M3 assesses the impact of predisposing, enabling and need factors on the utilisation of MHS. As a predisposing factor, age was the only significant predictor of MHS utilisation, with the utilisation rate among the 60–69 age group being merely 60.5% of that in the ≥ 80 age group. Except the variable of “live with children”, all other enabling factors had a significant influence on MHS utilisation. Older adults who received family support, had lower income, lived in rural communities, or participated in the urban-rural residents’ basic endowment insurance, were more prone to use MHS. Likewise, awareness of available MHS had a strong positive effect on utilisation, with respondents who recognized MHS availability being 5.122 times more likely to use such services. Two need factors, chronic diseases and self-rated health status, were significantly linked to MHS utilisation. Surprisingly, older adults with any chronic disease decreased the likelihhod of using MHS by 65.9%, while the utilisation rates of MHS among seniors with good or average self-rated health declined by 31.8% and 39.9%, respectively.

M4 examines the relationship between predisposing, enabling and need factors with the utilisation of SCS. Unlike the other models, none of the predisposing factors had a significant correlation with SCS utilisation. However, four enabling factors, including family support, self-assessed economic status, community type and social security benefits, emerged as significant predictors of SCS utilisation. Older adults who received family support were 2.640 times more likely to engage in SCS, while those with lower incomes or lived in rural communities reported higher levels of SCS utilisation. Similarly, awareness of available services was positively related to their engagement in SCS, with the likelihood of using the services being 99.209 times higher than those who lacked awareness. Of all need factors, only chronic illness had a significant impact on SCS utilisation, with older adults suffering from chronic diseases being more likely to participate in SCS programs.

## Discussion

All in all, the utilisation of HCBS was relatively low, as observed in previous studies. For instance, Wang [16] found that apart from 6.01% of older people who had actually used home medical visit services, the utilisation rates of other HCBS remained below 2.5%. Cao [29] further demonstrated that 59.59% of home-based disabled elderly in urban areas and 49.53% in rural areas have not received adequate support to manage their daily living and health needs. This trend, however, contrasts sharply with the rapid growth of the ageing population and their increasing demand for HCBS to facilitate ageing in place. According to Zhang and Li’s study [30], based on an analysis of tracking data, while there has been a significant reduction in the supply-demand gap for MHS, the gap for SCS has not noticeably narrowed, and the gap for LCS has even expanded to increase by 9% from 2005 to 2014. Furthermore, Lu and Zhou [31] found that the provision of HCBS often lacks an accurate assessment of older adults’ specific needs, leading to mismatches between supply and demand and an imbalance in the service structure. In reality, current policies may not sufficiently address key barriers to HCBS, such as accessibility, affordability, and awareness. Besides, substantial differences were found in the utilisation rates of LCS, MHS and SCS. By comparison, MHS received the most attention with the highest utilisation rates. Despite significant variation in health conditions, the majority of older adults sufferred from at least one chronic disease that required ongoing health knowledge and medical care, which likely explains the higher demand for MHS [32]. LCS ranked second in utilisation, as they in general serve as tools to compensate for the loss of self-care capability. When older people experience a decline in their functional abilities, especially those in the absence of family support, they have a tendency to seek LCS that can satisfy their essential demands for daily activities [33]. Conversely, SCS had the lowest utilisation rate, which might be attributed to the fact that older people often perceive emotional care as less critical. Many prefer to seek psychological support from family members rather than external organizations, resulting in lower demand for such services. As a whole, these different utilisation patterns highlight the distinct roles that MHS, LCS and SCS play in supporting older adults to live independently at home, while the uneven distribution of these services reveals significant gaps in the community care system. To address these disparities, there is a pressing need to expand and integrate HCBS services within community-based care system, ensuring that MHS, LCS, and SCS are readily accessible to meet the diverse and growing needs of the ageing population.

There were both substantial similarities and differences in the factors predicted by the Andersen Behavioral model that affected the utilisation of overall HCBS and its three specific service categories of LCS, MHS and SCS. Among these factors, age was found to be a key determinant of HCBS, LCS and MHS utilisation. This reflects a rising demand for services, particularly among middle- and oldest-old groups. These groups typically rely more on local government-provided services, as they face greater functional limitations and health challenges. This finding aligns with previous studies, such as Calsyn and Winter [9], Cox [34], and Crowell et al. [35], which emphasize the crucial role of government interventions in elderly care rather than personal purchases of services. Given this reliance on publicly funded HCBS, it is essential to ensure continued investment in these services to maintain accessibility and affordability for older adults. When individuals grow older with more complex care needs, they tend to seek LCS and MHS but often overlook SCS, possibly due to a lack of awareness and reluctance to seek help for mental health issues. However, older people are at heightened risk of loneliness and social isolation because of factors like chronic illness, mobility limitation, and the loss of family members [36, 37]. Therefore, local government should adopt an equity-based and person-centred approach in HCBS delivery. This should include education and training programs designed to improve awareness among older adults and their caregivers about mental health needs and the importance of SCS.

Family support emerged as a key factor in increasing the utilisation of HCBS, MHS and SCS, but not LCS, as shown in other relevant research [33]. Often, children give more attention to their older parents’ daily living needs rather than their medical and mental health needs. In this regard, informal family caregiving plays a substitute role for formal LCS, as many families provide direct assistance with daily activities such as meal preparation, cleaning, and mobility support. However, this reliance on family caregiving has resulted in a greater depandency on community-based MHS and SCS, which are primarily provided by local community. In line with the findings of studies by Calsyn and Winter [9] as well as Stoller and Cutler [11], this study found that income growth was associated with reduced utilisation of overall HCBS and its three categories. In China, wealthier older adults tend to bypass community-based services in favor of hiring private caregivers or relocating to luxury care homes when their care needs escalate [29, 38]. Such trend highlights a significant disparity in service access based on income level, which may undermine efforts to ensure equitable access to care for all elderly individuals. To promote healthy ageing in place, there is an urgent need to expand and redesign community-based HCBS to address various levels of care needs among older population.

In general, older people’s perceptions of HCBS, LCS, MHS and SCS provisions play a crucial role in determining service access. Raising awareness and ensuring equitable availability of elderly care programs and services within communities is essential to improve HCBS utilisation. Older adults with chronic diseases often pay more attention to keep a healthy lifestyle and establish daily routines, which can consequently lead to lower utilisation of LCS. However, their lower LCS utilisation does not indicate a decreased need for esential MHS and SCS. As supported by previous research, including Calsyn and Winter [9], Stoddart et al., [10] and Stoller and Cutler [11], health status was strongly linked to an older adult’s ability to access and use HCBS. Compared with other services, older people in poor health were more inclined to engage with MHS, as these services are crucial for managing chronic illnesses and providing ongoing health support. Meanwhile, self-care capability was found to have a significant impact on HCBS utilisation. As showned in Tables 5 9.2% of older adults with self-care limitations utilised LCS, compared to only 11.7% of those with full self-care capability. This disparity highlights that the demand for daily life assistance is largely determined by an indiviudal’s ability to perform essential activities independently. Therefore, policies should focus on improving the accessibility and affordability of both MHS and LCS, particularly for older adults with chronic conditions and limited self-care capabilities.

Community type played a significant role in determining older people’s access to HCBS, with notable differences in the utilisation patterns of LCS, MHS, and SCS between urban and rural areas. Urban communities had a larger share of LCS utilisation, possibly because of better infrastructure, wider service availability, and well-developed support networks compared to rural areas. By contrast, urban seniors had less access to MHS, as evidenced by Yang, Wang and Dai [39]. This is likely due to negative experiences with local medical services or a lack of confidence in their quality [40]. As a result, urban seniors tended to seek more reliable MHS at higher-level healthcare facilities. This indicates that urban primary medical services may either be underdeveloped or insufficient to meet the complex healthcare needs of ageing populations. There is a pressing need to diversify MHS offerings and improve specialised medical care that are universally accessible across both urban and rural regions. In addition, urban residents, benefiting from a broader range of leisure and recreation activities, were predicted to have higher levels of social participation than their rural counterparts [41]. This greater social engagement have presumably reduced their reliance on SCS, contributing to the lower utilisation rate of these services in urban areas. Accordingly, the overall utilisation rate of HCBS remained generally lower in urban than rural settings, which aligns with Peng, Song and Huang’s study [25]. Similarly, urban employees’ basic endowment insurance was evaluated to have a positive effect on LCS utilisation but a negative influence on MHS utilisation. These disparities not only highlight that the more severe challenges of elderly care in rural areas, but also indicate a selective abandonment of basic care services by urban older individuals, who may prefer private or higher-tier healthcare options over community-based services. Indeed, rural communities face significant challenges with both the availability and quality of elderly care services, making it more difficult for older adults to receive adequate support. It is of most importance for policymakers to establish an integrated elderly care system that guarantees equitable access to and choice of HCBS for older adults in rural areas.

**Table 5:** Crosstabs between self-care capability and the utilisations of HCBS, LCS, MHS, and SCS

	Utilisation of HCBS		Utilisation of LCS		Utilisation of MHS		Utilisation of SCS	
	Yes	No	Yes	No	Yes	No	Yes	No
**Self-care capability**								
Impaired self-care	42 43.8%	54 56.3%	28 29.2%	68 70.8%	31 32.3%	65 67.7%	17 17.7%	79 82.3%
Full of self-care	338 31.4%	737 68.6%	128 11.7%	949 88.3%	282 26.2%	793 73.8%	69 6.4%	1006 93.6%

### Contributions and limitations

This study applies the Andersen Behavioral model to analyse the utilisation of overall HCBS and its three specific service categories of LCS, MHS and SCS. It identifies both similarities and differences in the factors influencing the utilisation of these services, in order to provide valuable insight to guide policy development aimed at better match of demand with supply. By expanding the application of the Andersen behavioral model to different aspects of elderly healthcare in China, this study offers a nuanced understanding of the complexities of service utilization within the context of an ageing population. In addition, the research findings shed light on the significant role of predisposing, enabling and need factors in shaping the utilisation patterns of LCS, MHS and SCS. These insights provide practical guidance for optimising the healthcare system and reshaping HCBS delivery, ensuring that services are better tailored to the specific needs of older adults.

Although this study contributes to a better understanding of different influences on HCBS utilisation, there were some limitations. Firstly, this study selected samples from JJ District in Zhejiang Province, where urban and rural communities have a comparative advantage and are able to provide a range of elderly care services. Caution should be taken when generalising these findings to less developed areas, where both the availability and quality of services may differ. Secondly, this research focused primarily on LCS, MHS and SCS, it did not cover the full spectrum of community services. Further research is needed to examine demand, supply and utilisation from a wider variety of elderly care services, so that more holistic and inclusive policy actions can be introduced. Thirdly, due to data limitations, no variables reflect emotional and cognitive processes in the need factors, which might impact the research findings.

## Conclusions

China has seen a rapidly ageing population, and the 9073 or 9064 strategy highlights the growing importance of HCBS in supporting 90% of older people to age at home. However, despite this policy emphasis, the utilisation rates of overall HCBS as well as the three typical services of LCS, MHS and SCS remained relatively low. The factors influencing HCBS utilisation exhibited both similarities and differences. Young seniors tended to use LCS and MHS at lower rates compared to their older conterparts. In contrast, those with family support or chronic diseases were more likely to utilise MHS and SCS. Additionally, financial constraints and urban-rural disparities continue to shape older adults’ access to all three services. There is an urgent need to develop a community-based integrated care system, in which each type of HCBS is clearly defined in terms of its respective and complementary role in supporting healthy ageing in place. To achieve this, both national and local governments should make a commitment to build high-quality, equity-based, and person-centered services, working collaboratively with families to ensure comprehensive and inclusive support for older adults. This approach is essential improving medical health outcomes while also enhancing spiritual well-being among ageing population. Urban-rural disparities in the utilisation of HCBS, LCS, MHS and SCS require a more balanced and equitable development of welfare services across all regions. Addressing these disparities is critical to support older people maintain their independence for as long as possible.

## Data Availability

The data presented in this study are available from the corresponding author on reasonable request. The data are not publicly available due to their containing information that could compromise the privacy of research participants.

## Abbreviations

HCBS: Community home-based care services
LCS: Life care services
MHS: Medical health services
